# The Role of Platelet Concentrates and Growth Factors in Facial Rejuvenation: A Systematic Review with Case Series

**DOI:** 10.3390/medicina61010084

**Published:** 2025-01-07

**Authors:** Giuseppina Malcangi, Angelo Michele Inchingolo, Alessio Danilo Inchingolo, Laura Ferrante, Giulia Latini, Irma Trilli, Paola Nardelli, Marialuisa Longo, Andrea Palermo, Francesco Inchingolo, Gianna Dipalma

**Affiliations:** 1 Department of Interdisciplinary Medicine, University of Bari “Aldo Moro”, 70124 Bari, Italy; giuseppinamalcangi@libero.it (G.M.); ad.inchingolo@libero.it (A.D.I.); lauraferrante79@virgilio.it (L.F.); dr.giulialatini@gmail.com (G.L.); trilliirma@gmail.com (I.T.); drnardellipaola@gmail.com (P.N.); dott.marialuisa.longo@gmail.com (M.L.); giannadipalma@tiscali.it (G.D.); 2 Department of Experimental Medicine, University of Salento, 73100 Lecce, Italy; andrea.palermo@unisalento.it

**Keywords:** PRP, facial esthetic, photoaging, rejuvenation, skin, growth factors, regenerative medicine, wrinkles, skin degradation, skin aging

## Abstract

*Background and objectives*: Due to the regeneration potential of growth factors (GFs) and platelet concentrates (PCs), facial rejuvenation has been a major area of attention in esthetic medicine. The effectiveness and safety of PCs and GFs in promoting face rejuvenation are examined in this systematic review, which is complemented by a case series. GFs are essential for collagen production and dermal matrix remodeling, while PCs, like Platelet-Rich Plasma (PRP), are abundant in bioactive chemicals that promote tissue healing and cellular regeneration. *Materials and Methods*: A comprehensive literature search was performed on PubMed, Web of Science, and Scopus, focusing on human clinical trials published between February 2019 and February 2024 related to PRP and facial esthetics. *Results*: Thirteen studies met the inclusion criteria and were analyzed. *Conclusions*: The review summarizes the most recent data on patient outcomes, treatment regimens, and possible hazards. The case series that goes with it shows real-world examples of how to improve skin elasticity, texture, and general facial appearance with little negative side effects. These results highlight the potential use of PCs and GFs as minimally invasive procedures.

## 1. Introduction

The ‘healthy and youthful appearance of the skin is closely determined by a system of balance between anabolism and catabolism of extracellular matrix (ECM) proteins. The ‘progressive alteration of these metabolic processes results in decreased microvascularization, reduced production of collagen and elastin, increased degradation of them, and consequent production of thinner and disorganized fibers [1]. This imbalance results in the appearance of wrinkles, furrows, altered pigmentation, loss of elasticity, texture, and skin radiance, better defined as skin aging [2,3,4].

Two biologically distinct aging processes are recognized: intrinsic (reduction in hydration, thinning of the dermis due to loss of collagen, and degradation of the elastic fiber network) and extrinsic (environmental factors, photoaging due to exposure to ultraviolet rays) [5,6,7,8].

In both aging processes, there is an increase in matrix metalloproteinases (MMP)-1, MMP-2, and MMP-9 that cause degradation of dermal connective tissue fibers and matrix proteins [9,10,11,12].

Photoaging by ultraviolet (UV) radiation manifests a greater presence of MMPs with more pronounced connective tissue alterations, given by a greater space present between connective tissue fibers and a greater reduction in vascularization, the latter probably due to the damage and thus the difficulty of the connective tissue no longer being able to maintain the normal structure and function of the vascular system in the damaged skin [13,14,15,16,17].

Understanding the biological mechanisms of changes in skin angiogenesis and photoaging skin processes, thinking about using a therapeutic agent that can block degradation processes and activate regenerative processes, could promote esthetic regenerative treatments of the face and beyond [18,19,20,21,22].

Considering the tissue rejuvenation process comparable to the metabolic processes triggered in wound healing processes, autologous platelet concentrates (PC) were considered [23,24,25,26,27,28,29,30,31,32,33].

The concentration of platelets in the human blood of a healthy patient is 150,000–350,000/microliter. PCs contain about 1,000,000 of them per microliter in a minimal volume of plasma [34,35].

PCs and autologous growth factors (AGFs), obtained by different processes and techniques of centrifugation of patient blood sampling, have been considered to slow down skin aging processes and stimulate cellular activity, improving the esthetics of the face, neck, decolletage, and even skin of the hands, areas more easily marked by aging [18,36,37,38,39]. The use of PCs and AGFs has evolved in use over the years. The first application was used in maxillofacial surgery in 1994, in the form of platelet gel (referred to as Toyapongsaki) in maxillofacial surgery as a bone mass thickener in a mandibular excision, to be later applied in many branches of specialty medicine [40,41,42,43].

To date, several forms of GF use are known and have been refined over the years. Modifying preparation methods, based on leukocyte concentrations and on ‘fibrin architecture, the different categories are distinguished: fibrin glue (Tissucol Baxter 1994), Platelet-Concentrated cPRP (Marx-Garg 1998), Platelet-Rich Plasma (PRP), Plasma Rich in Growth Factor (PRGF 1998 Anitua), Platelet-Rich Fibrin (PRF 2001 J. Choukroun) to the best-performing PCs in regenerative medicine with Concentrated Growth Factors (CGF Corigliano et al., 2006), Liquid Phase Concentrated Growth Factors (LPCGF) and Activated Plasma Albumin Gel (APAG) [44,45,46,47].

The reparative function of platelets has long been known through the degranulation of alpha granules, which are considered the most important growth factors (GF), such as platelet-derived growth factors (PDGFaa, PDGFbb, PDGFab), endothelial growth factor (VEGF), transforming growth factor β1 and -β2 (TGF-β1 and TGF-β2), TNF-α, basic fibroblast growth factor (bFGF), and epithelial growth factor (EGF), all of which play an important role in the early phase of the wound healing process, accelerating it [48,49,50,51].

**Figure 1:**
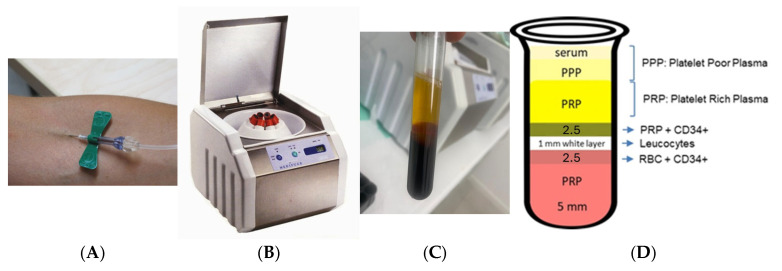
PRP preparation process. Venous blood is taken from the patient (**A**) and centrifuged with the Medifuge 200 device Silfradent ^®^ (**B**) to divide it into three distinct layers (**C**,**D**): red blood cells (RBC) at the base, PRP at the top, and the buffy coat in between. This illustration provides a rough breakdown of cell types within the buffy coat, emphasizing the arrangement of neutrophils, eosinophils, basophils, monocytes, lymphocytes, and platelets [55,56].

In the scientific literature, there is evidence of the multiplicity of PRP preparation techniques, administration intervals, and sites treated with a finding of varying results between them, although, in general, its use manifested improvement in the parts of the face, hands, and decollete treated (Figure 1) [34,52,53,54].

PRF is an evolution of PRP and PRGF, in solid (gel) or liquid form, obtained by a single centrifugation without the addition of anticoagulants or platelet activators, hence completely autologous [57,58,59,60]. Lower relative centrifugal force (RCF), shorter centrifugation time, and the use of plastic tubes create an injectable liquid product (I-PRF) capable of capturing more platelets and leukocytes that, by remaining trapped in the gelled fibrin matrix after inoculation, can slowly release GF [48,61,62,63,64].

Leukocytes also stimulate fibroblast propagation, enhance anti-inflammatory effects, angiogenesis, and production of proteins (e.g., procollagen) necessary for structure and remodeling of the extracellular matrix [46,65,66,67].

The bioregenerative results of both objective and subjective areas of facial skin treated with PRF are found to be very high performing with a reduction in wrinkles and pigmentation and increased skin brightness and elasticity [68,69,70,71,72,73,74,75,76].

The latest tissue bioengineering studies have focused on evaluating the speed, time, and ‘angle’ of centrifugation of venous blood sampling to obtain a product with greater reparative and regenerative predictability [77,78,79,80].

PPP is the top part of the centrifugate and contains a much higher amount of fibrinogen, albumin, white blood cells, and soluble cytokines [81,82,83,84]. Its use in esthetic medicine manifested immediate and lasting effects on fine wrinkles, with greater efficacy on dark circles and infraorbital folds by acting as a scaffold for the sustained release of GF than PRP, which, by stimulating the release of fibroblasts and acting as an anti-inflammatory, improved skin smoothness and texture [85,86,87,88].

**Figure 2:**
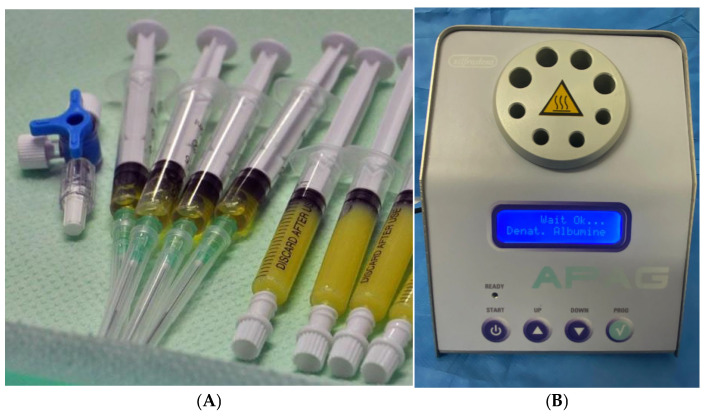
APAG on the right and PRP with CD34+ on the left for autologous filler preparation (**A**); APAG Silfradent^®^ (**B**).

Heating PPP (2 mL) to 75 °C for 10 min with a dedicated frequency heater (APAG Silfradent^®^) produces gelled PPP, which, when mixed with CD34+ (0.5 mL) and PRP (0.5 mL), produces APAG, an autologous filler enriched with stem and GF. APAG produces better-performing results, combining the immediate smoothing effect of the gel on wrinkles with prolonged cellular activation over time due to the presence of stem and GF (Figure 2) [89,90,91,92].

The technique on the use of PC and GF, derived from autologous products, appears to be safe. The appearance of small hematomas, the use of needles for product inoculation or microneedling, appear to be the only aspects of discomfort for the patient [93,94].

The purpose of this work was to be able to evaluate the use of GFs and PCs as a viable therapeutic choice in skin rejuvenation [95,96,97].

Unfortunately, more clinical studies are needed, as scientific evidence on the use of PCs AND AGFs is currently very limited [98,99,100,101,102,103,104].

The study addresses skin aging caused by the imbalance in ECM protein turnover, leading to collagen degradation, loss of elasticity, and wrinkles. It highlights the potential of platelet concentrates (PC) in regenerative esthetics to improve skin quality and counteract aging effects. The study explores how PCs stimulate collagen production, enhance elasticity, and support dermal regeneration. It also emphasizes standardizing preparation protocols to improve clinical outcomes and address gaps in current anti-aging treatments.

## 2. Materials and Methods

### 2.1. Protocol and Registration

This systematic review was conducted by the standards of the Preferred Reporting Items for Systematic Reviews and Meta-analysis (PRISMA) 2020 statement [105,106,107,108]. The protocol of the review was registered at PROSPERO under the unique number 601404.

### 2.2. Search Processing

The search period started on 12 August 2024, and the last search was carried out on 25 September 2024.

“**PRP**”, “**Facial Aesthetic**”, “**Photoaging**”, and “**Rejuvenation**” were the search terms utilized on the databases (PubMed, Web of Science, and Scopus) to select the papers under evaluation, with the Boolean operators “AND” and “OR”. Only content published in English over the previous five years (February 2019 to February 2024) was included in the search (Table 1).

### 2.3. Eligibility Criteria

Working in pairs, the reviewers selected pieces that met the following requirements to be included: (1) research involving just human beings; (2) clinical studies; and (3) our most relevant case series about skin aging and scars.

One of the exclusion criteria was in vitro research. The following were also included: (1) research on animals; (2) case reports; and (3) narrative reviews, meta-analyses, and systematic reviews.

Duplicate studies were removed manually.

### 2.4. Data Processing

Working separately, two reviewers (I.T. and A.D.) used the predetermined inclusion and exclusion criteria to filter the data that was taken from each database. Individual choices were hidden from the researchers. Both reviewers’ results were converged upon in the final meeting. The complete text was obtained and examined when a reviewer thought an article might be accepted. Both independently and twice, this occurred.

Each qualifying main study’s authors and publication date, study type, purpose, materials and methods, and findings are among the data that were taken from it.

Reviewers’ disagreements on which article to choose were resolved through discussion.

### 2.5. Quality Assessment

Two reviewers, G.L. and F.I., evaluated the included papers’ quality using the reliable Cochrane risk-of-bias assessment for randomized trials (RoB 2). This test assesses six potential areas of bias: inadequate outcome data, selective reporting, blinding of participants and staff, random sequence generation, allocation concealment, and outcome assessment blinding. A third reviewer (L.F.) was consulted if there was a disagreement and continued until a consensus was reached.

## 3. Results

**Figure 3:**
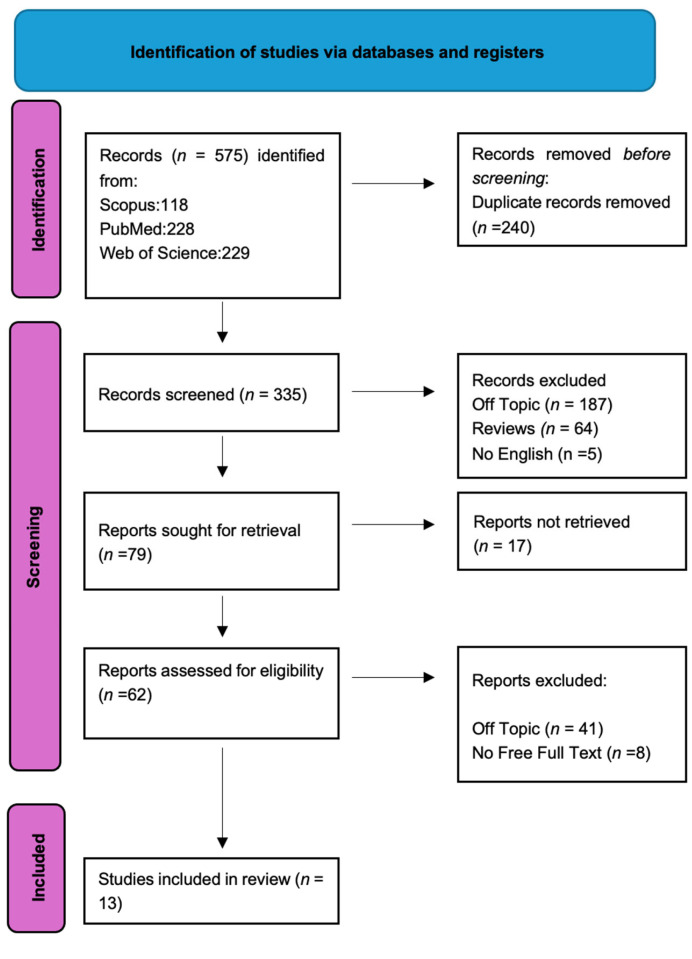
PRISMA flowchart diagram of the inclusion process. The literature search’s Preferred Reporting Items for Systematic Reviews and Meta-Analyses (PRISMA) flow diagram.

Keyword searches of the Web of Science (229), Scopus (118), and PubMed (228). A total of 575 articles were found in the databases. After the duplicates were eliminated (240), 335 articles were included. In addition, 322 of these 335 studies were disqualified for violating the inclusion criteria that had been previously established. After screening, thirteen papers were chosen for this work (Figure 3). Each study’s findings were listed in Table 2.

In total, seven studies were randomized, and nine studies were non-randomized. This includes randomized clinical trials, randomized controlled trials, and non-randomized observational studies.

The minimum group size was 10 participants, as seen in studies like the one by Tsai et al. (2024) [109] with 10 participants. The maximum group size was 94 participants, as reported in studies such as Ulusal B. (2017) [110], which involved 94 female patients.

The most common reasons for therapy across the studies include facial skin rejuvenation, photodamage, and aging-related skin conditions. PRP (Platelet-Rich Plasma) was most frequently used to treat issues like wrinkles, skin texture, and overall skin vitality, with the goal of improving facial appearance and reversing signs of photoaging.

PRP (Platelet-Rich Plasma) was the most frequently used therapeutic agent across the studies, often in combination with other treatments like hyaluronic acid (HA) or saline solution. In some studies, other treatments such as ADSC (Adipose-Derived Stem Cells) and PPP (Platelet-Poor Plasma) were also evaluated.

Some common limitations identified in the studies include small sample sizes, lack of standardization in PRP preparation and application, variability in treatment protocols, and limited follow-up periods. There were also issues with inconsistent results across different studies, making it difficult to generalize findings. Errors related to subjective assessments, such as patient satisfaction or physician evaluations, and the absence of long-term efficacy data were also noted as limitations.

**Table 2:** Qualitative analysis of the studies included.

Authors	Type of the Study	Patients	Matherial and Methods	Aim of the Study	Conclusions
Hassan et al. (2020) [111]	Prospective, uncontrolled	11 females	Monthly intradermal injections of injectable i-PRF for 3 months in specified facial areas.	To evaluate the efficacy of i-PRF for facial skin rejuvenation using objective skin analysis and patient-reported outcomes.	Significant improvements in skin spots and pores; increased patient satisfaction.
Draelos et al. (2020) [112]	Pilot study	20 subjects (30–60 years, both genders)	PRP in a preservative serum, applied twice daily for 8 weeks after electroporation.	Evaluate PRP serum effects on facial photoaging.	PRP showed 90-day stability; improved rete peg architecture and collagen I expression.
Rina Du et al. (2020) [113]	Clinical study	30 females (30 to 50 years)	Autologous PRP injected three times at 15-day intervals, analyzed with VISIA^®^ and organotypic skin models.	Investigate the molecular mechanisms of PRP in rejuvenating aged skin.	PRP improved skin quality, reducing wrinkles and photoaging markers.
Gawdat et al. (2024) [114]	Split-face randomized study	20 females (35 to 55 years)	PRP on one side, GF on the other; assessed with GAIS and OCT.	Compare PRP and GF for skin rejuvenation.	Both improved skin vitality; PRP had better long-term results and satisfaction.
Lee et al. (2023) [115]	Pilot study	31 participants (27 females, 4 males) aged 27 to 71 years (median age: 38)	PRP treatment, evaluated using the WSRS and GAIS.	To evaluate the effectiveness and patient satisfaction of PRP treatment for photodamaged skin.	Modest benefits in skin aging treatment; adverse effects mild (e.g., tenderness, swelling).
Charles-de-Sá et al. (2020) [116]	Comparative experimental study	20 human subjects with aged or photoaged skin.	Two different therapies: PRP injections and expanded ADSC therapy.	To compare the effects of PRP and ADSC therapy on aged human skin.	PRP did not produce significant tissue regeneration. ADSC treatment was linked to ECM remodeling, new elastic fiber production, and the degradation of elastotic material.
Murad Alam et al. (2018) [117]	Randomized clinical trial	27 participants aged 18–70, of which only 19 completed the study	Each participant received 3 mL of PRP in one cheek and saline in the other. Follow-up included digital photos at baseline, 2 weeks, 3 months, and 6 months.	To assess PRP’s effect on photoaging and compare it with normal saline (as a placebo).	PRP did not result in a significant improvement in photoaging scores when compared with normal saline based on independent dermatologist assessments of standardized photographs.
Paweł Surowiak et al. (2023) [118]	Prospective non-randomized controlled clinical study	10 volunteers (5 women and 5 men) between the ages of 29 and 49	PRP versus placebo (NaCl). After 21 days, skin biopsies were performed.	To test whether PRP stimulates procollagen type I synthesis in human skin compared with placebo.	PRP increased the expression of procollagen type I compared with placebo.
Betul Gozel Ulusal (2016) [110]	Observational clinical study	94 female patients, average age 53.0 ± 5.6 years.	PRP kit (Dr B PRP™) and centrifuge. Hyaluronic Acid (HA) gel (3.5%) and procaine.	To evaluate the effectiveness of a combined treatment of PRP and HA for facial rejuvenation.	A combination of PRP and HA injections, with additional needling, resulted in significant facial rejuvenation.
Tsai Y. et al. (2024) [109]	Double-blind randomized controlled splitting face study	10 participants	The participants were randomly assigned to receive 2.5 mL injections of PRP and PPP on different sides of the face in three sessions with 1-month intervals.	Compare the efficacy of PRP and PPP for facial rejuvenation.	PRP and PPP are effective in treating facial photoaging. PRP exhibited slightly superior efficacy in enhancing overall skin condition, while PPP was slightly more effective in improving shallow wrinkles.
Everts P. et al. (2019) [119]		Eleven healthy female volunteers between 45 and 65 years old	All women signed an informed consent form before treatment with 3 facial PRP injections.	the efficacy of autologous PRP injections for facial skin rejuvenation.	A series of 3 Pure PRP injections at 6-month follow-up resulted in significant skin rejuvenation as demonstrated by biometric parameters and confirmed by patient self-assessment score.
De Silva L. et al. (2021) [120]	Non-randomized, controlled, pilot trial	Nineteen women (54 years ± 7 years) with Glogau photoaging II and III types	All patients received monthly intradermal injections of lyophilized PRP and saline solution (as a control) into the facial skin during a period of 2 months.	Evaluate the effect of lyophilized PRP in the treatment of skin aging.	In a well-controlled pilot split-face study using VISIA ^®^ and SHG methods, we did not demonstrate an effect of lyophilized PRP by mesotherapy on skin aging.
Hui Q. et al. (2017) [121]		13 female patients suffering from facial aging	13 facial aging females were treated with ultra-pulsed fractional CO_2_ laser. One side of the face was injected with PRP as the experimental group, while the other, as the control group, received the same dose of saline.	Evaluate the potential synergistic effects of combining PRP with ultra-pulsed fractional CO_2_ laser for facial rejuvenation therapy.	PRP and ultra-pulsed fractional CO_2_ laser had a synergistic effect on the therapy for facial rejuvenation.

Note: Adipose-Derived Stem Cell (ADSC), hyaluronic acid (HA), sodium chloride (NaCl), Platelet-Rich Plasma (PRP), Platelet-Rich Fibrin (i-PRF).

### Case Series

In the Case Study section, images of the study participants are presented. Informed consent for the publication of these images was obtained from all participants. Additionally, the study was approved by the relevant ethics committee, which included consent for the use and publication of participant images.

**Figure 4:**
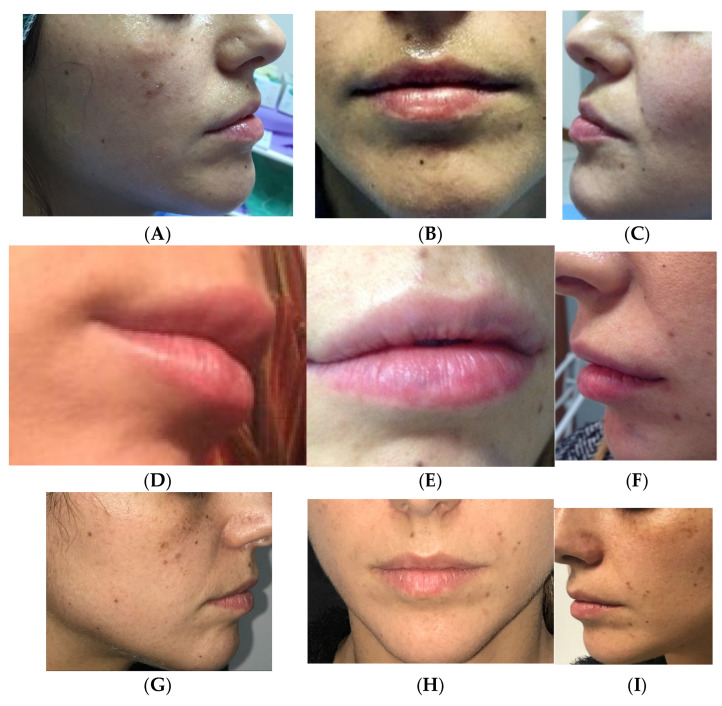
Before treatment (**A**–**C**), immediately after the first treatment (**D**–**F**), and after two years without other treatments (**G**–**I**).

Clinical case 1. A 26-year-old female patient, in good health, with a request for volume augmentation of the upper and lower lips. The treatment was performed with lip filler with APAG (Figure 4).

**Figure 5:**
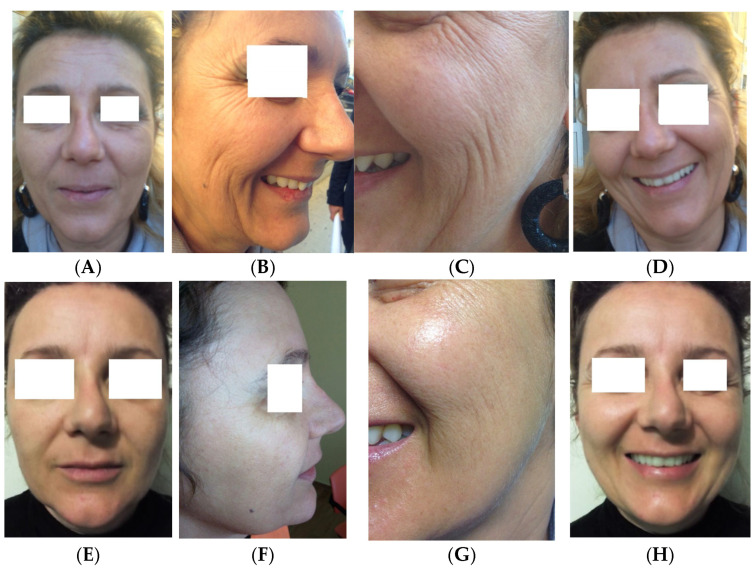
Before treatment, 13 December 2014 (**A**–**D**), and after three treatments, 24 March 2015 (**E**–**H**).

Clinical case 2. Healthy female patient aged 45 years with a request for antiaging treatment, with repair of wrinkles and full-face skin texture. The treatment was filler with APAG and LP CGF for the lips and zygomatic area and mesotherapy with LP CGF full-face (Figure 5).

**Figure 6:**
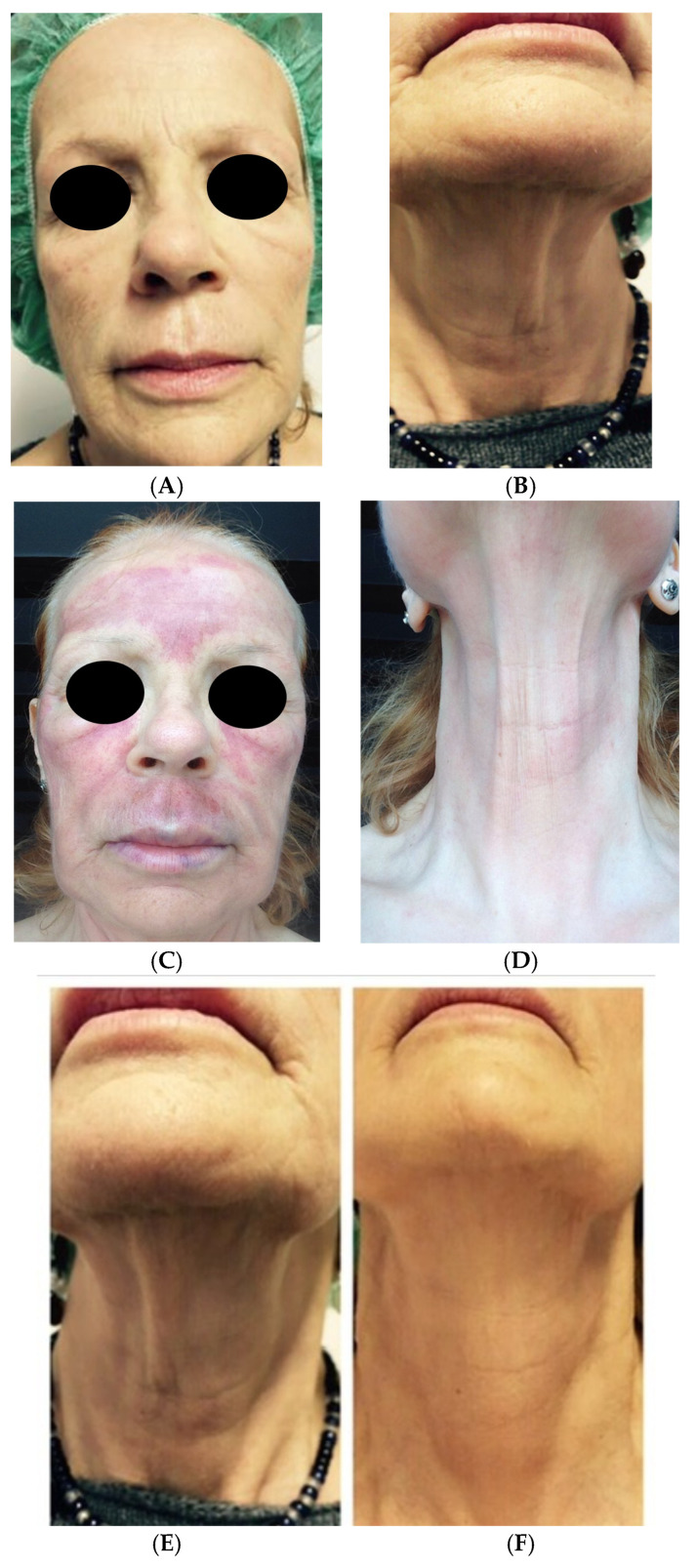

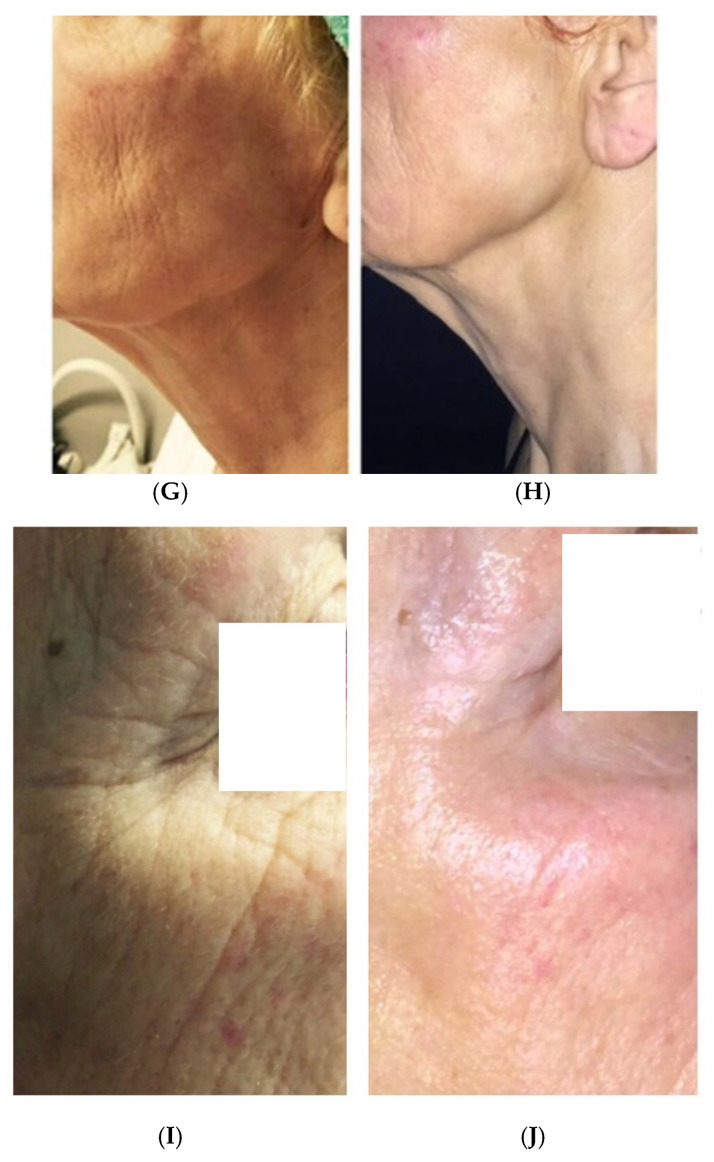

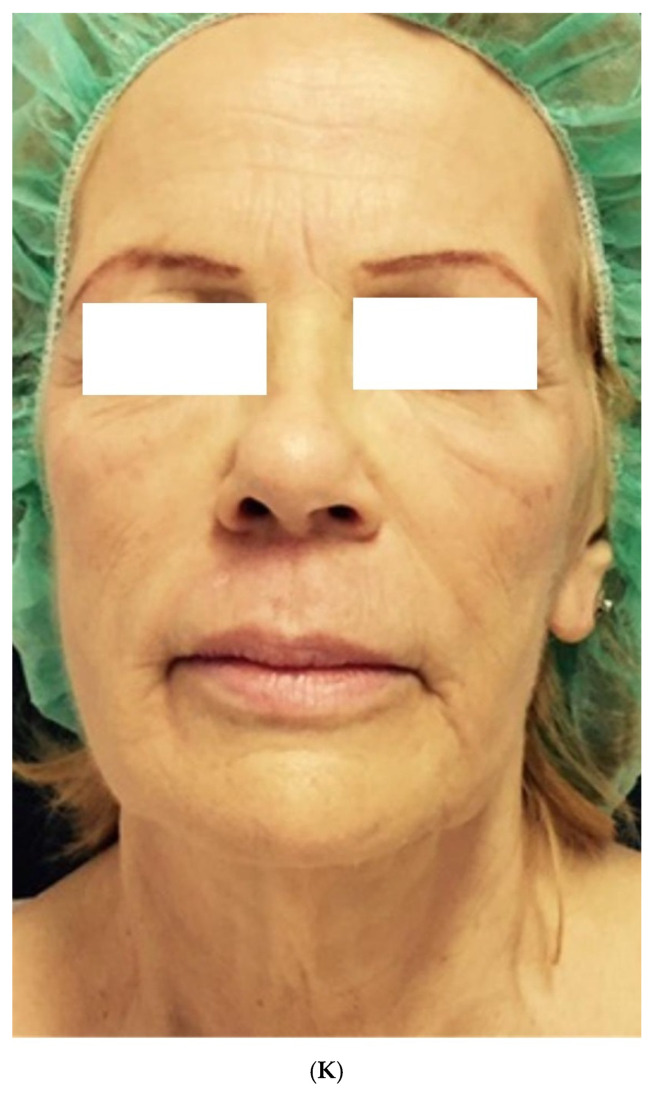
Before (**A**,**B**) and immediately after the treatment (**C**,**D**) of LPCGF with microneedling. The desired esthetic effect was best achieved through a noticeable reduction in signs of aging and a general improvement in skin texture. In comparison: signs of skin aging in the neck, cheek, and cheekbone area before treatment (**E**,**G**,**I**) and the clear esthetic improvements in the same areas after treatment (**F**,**H**–**J**). Desired final esthetic result achieved of face and neck (**K**): improvement of skin texture and reduction in wrinkle depth.

Clinical case 3. A 72-year-old female patient in good health with a request for antiaging treatment to resolve skin relaxation in the face and neck. The treatment was filler with APAG and LP CGF for the lips and zygomatic area and mesotherapy with LP CGF full-face (Figure 6).

**Figure 7:**
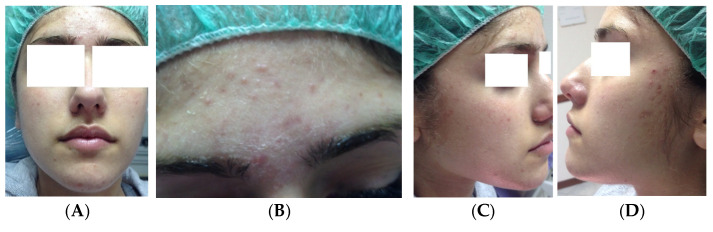

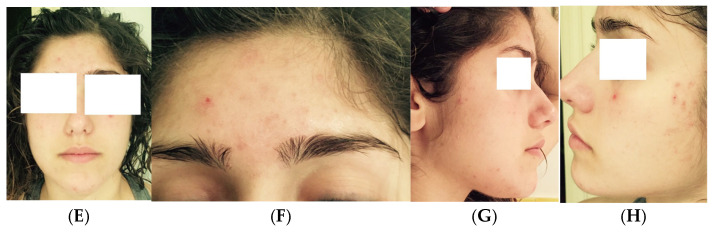
Before treatment (**A**–**D**) and 5 days after the first treatment (**E**–**H**).

Clinical case 4. A 16-year-old female patient in good health, requiring treatment for acne and acne scars. The treatment was CGF and microneedling (Figure 7).

**Figure 8:**
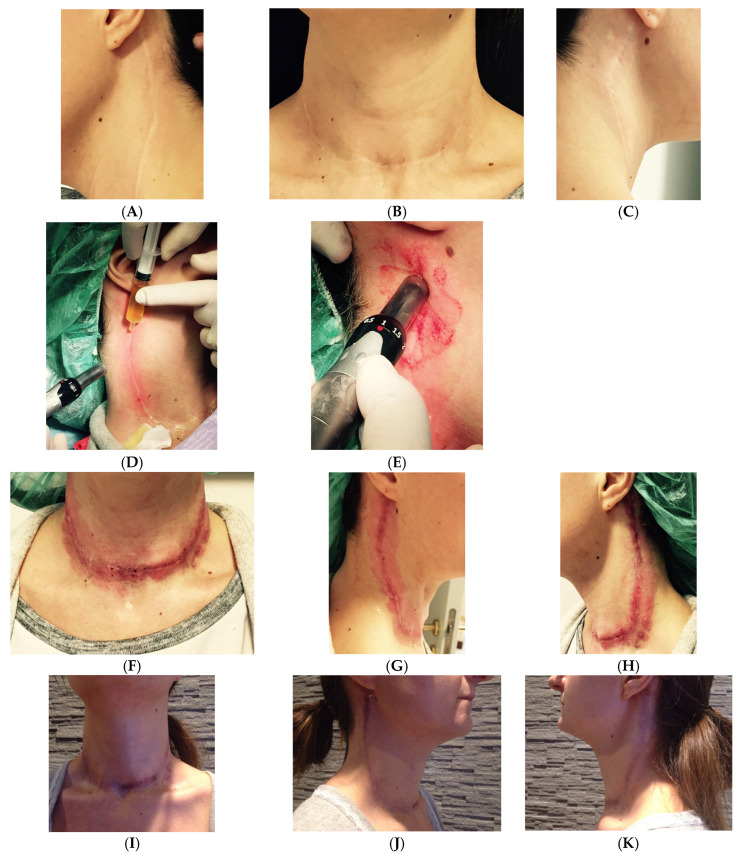

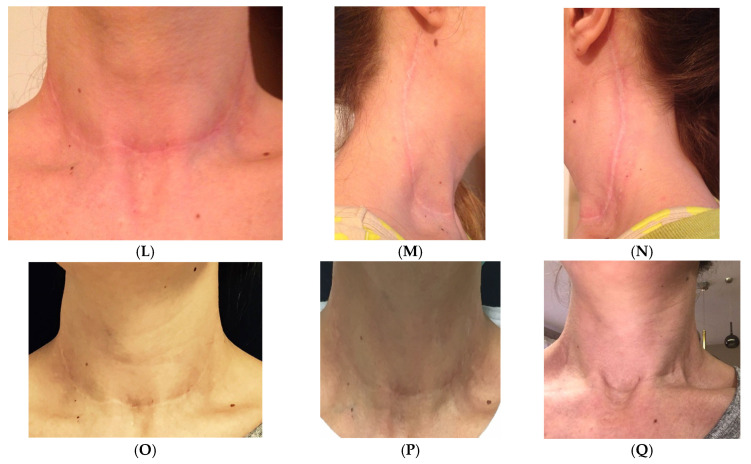
Before (**A**–**C**), during the first treatment (**D**,**E**), immediately after the first treatment (**F**–**H**), after three days (**I**–**K**), and after 10 days from the first treatment (**L**–**N**). Hyperemia and swelling of the scar area are expected consequences of surgery. Hyperemia, in particular, is due to the use of needling to a depth of 2/3 mm to treat scar fibrosis. These symptoms are temporary, normal, and part of the natural healing process. Over time, the hyperemia and swelling will gradually subside as the tissue heals (**D**–**H**). The same patient before (**O**), after three treatments (**P**), and after three years, without further treatment (**Q**).

Clinical case 5. Large post-surgical scar of thyroid carcinoma (40 cm) treated with LP CGF and microneedling in a 32-year-old female patient in good health (Figure 8).

**Figure 9:**
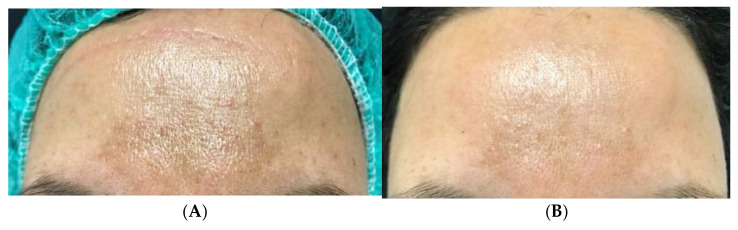

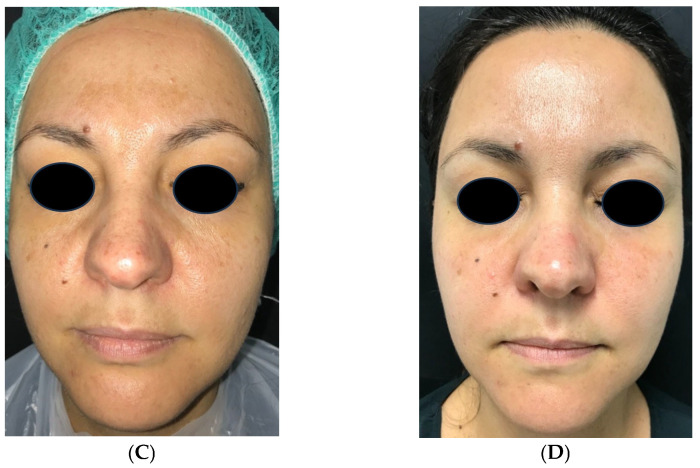
Before treatment (**A**,**C**) and after the second treatment, after 3 weeks (**B**,**D**).

Clinical case 6. A 42-year-old female patient, suffering from hypercholesterolemia treated with statins, with melasma, dark or hyperpigmented spots on the forehead and face, treated with LP CGF and microneedling full-face in two sessions 3 weeks apart (Figure 9).

## 4. Discussion

In recent years, PRP (Platelet-Rich Plasma) has gained popularity for its applications in the regenerative field, particularly in esthetic medicine and the treatment of skin aging. PRP is prepared using a patient’s autologous blood and contains platelet GF that stimulate tissue repair and collagen production [122,123,124,125,126]. However, recent research indicates that another formulation, PRF (Platelet-Rich Fibrin), may have greater regenerative potential due to its three-dimensional fibrin matrix that allows prolonged release of GF, promoting more effective tissue regeneration. Whereas PRP is prepared by rapid centrifugation that separates platelets without clots, PRF is formed using slow centrifugation without anticoagulants, which leads to the formation of a fibrin lattice [127,128,129]. This lattice traps GF and allows gradual and steady release, which is ideal for the long-term regenerative process. The characteristics of PRF have been well documented, for example, in the Hassan et al. study, where PRF showed improvements in skin texture and patient satisfaction in a more stable manner than PRP. PRF, because of its structure, not only promotes tissue healing but also the formation of new blood vessels and controlled inflammation, key elements for complete skin regeneration. In addition, recent studies have proposed a further evolution of PRF, CGF (Concentrated Growth Factors), which optimizes the concentration of GF and may represent one of the most promising alternatives in the regenerative field. Although PRP is a viable option for skin regeneration, PRF may offer additional benefits due to its fibrin matrix and sustained release of GF. This suggests that PRF, or even CGF, may become a preferred choice for long-term treatments requiring more robust and durable regeneration [130,131,132,133,134].

Other regenerative techniques with GF like LPCGF, APGF (Autologous Platelet and Growth Factors), and microneedling are innovative tools that naturally stimulate tissue repair and enhance esthetic and functional results [135,136,137]. These methods use the patient’s own biological materials to promote tissue regeneration, offering a synergistic approach that visibly improves appearance while supporting tissue health. LPCGF adds antimicrobial and anti-inflammatory benefits, making it ideal for complex healing scenarios. APGF further stimulates tissue renewal and improves skin texture, enhancing both recovery speed and quality in esthetic and post-surgical treatments. Microneedling, on the other hand, creates micro-perforations in the skin that boost collagen and elastin production, refining skin texture and complementing GF treatments for a maximized regenerative effect. Together, these techniques provide a natural, effective approach to comprehensive tissue rejuvenation [81,134,138].

### 4.1. PRP for Facial Rejuvenation

PRP has gained wide acceptance in the last few years as an instrument of treatment because of its non-invasive action for skin rejuvenation. It enhances the quality of the skin, minimizes wrinkles, and thus triggers regeneration of tissues. PRP, being prepared from the patient’s autologous blood, is rich in GF, which has a stimulatory effect on tissue repair and collagen production. Although there is already a fair amount of literature on its support, more research is necessary for the elucidation of standards of treatment and long-term results as well as comparison with other options of regenerative therapy [139,140,141].

One of the recent studies relating to this approach toward PRP was conducted by Hassan et al. They investigated the PRP variant (Platelet-Rich Fibrin) for skin spot improvement, skin texture, and patient satisfaction; they found that while changes in wrinkle reduction were not statistically significant, the skin spot improvement was excellent. Such outcomes suggest that other blood-derived treatments, like PRF, do in fact show similar yet not identical benefits when compared to PRP and reinforce a need for systematic comparisons among these methods [142,143,144].

For facial rejuvenation, the research made by Draelos et al. examined the innovative use of autologous PRP in a topical cream over an eight-week period with 20 healthy participants aged 30 to 60 years assessing the effects of a PRP-containing serum compared to a serum without PRP [145,146]. The study showed that PRP remained stable for 90 days when refrigerated, highlighting its potential for topical use, and histological analysis revealed enhanced skin structure with increased collagen type I levels after applying the PRP serum. Despite these findings, both the PRP serum and a non-PRP serum demonstrated significant improvements in skin texture, raising questions about the unique benefits of PRP in this context. The treatments were well tolerated, and electroporation was used to improve absorption, addressing a challenge in topical PRP application. Future studies should aim to extend treatment duration and explore long-term efficacy, as the results suggest potential for PRP in skin rejuvenation but need further validation [147,148,149].

At the molecular level, Du et al. investigated the mechanisms of the effects of PRP. The authors demonstrated that injections of PRP may minimize the injury from aging and photoexposure by enhancing the synthesis of such important structural proteins as fibrillin and tropoelastin. This in vitro model study further supported previous concepts about the activity of PRP in the protection of skin against the detrimental influence of UV rays and maintenance of epidermal structure [113,150,151].

An interesting comparison of PRP to ready-to-use GF was given by Gawdat et al. In this split-face study, where each side of a patient’s face received different treatments, it emerged that PRP yielded more long-lasting results than ready-to-use GF, with a higher level of satisfaction in the PRP-treated patients [152,153]. This finding points out the fact that even though PRP might take more time to prepare than GF, more benefits might be yielded by PRP [154,155].

In the pilot study by Lee et al., the effectiveness of PRP for treating photodamaged skin was evaluated with 31 participants aged 27 to 71 over a follow-up period of 5.7 weeks. The study found only one patient showing improvement based on the Wrinkle Severity Rating Scale, while the Global Aesthetic Improvement Scale indicated esthetic enhancement in 14 patients, although 17 patients saw no change. Despite this, patient satisfaction was high, with 74.2% pleased with the results, and 80% believing the treatment was worth it, feeling an average of 19.2 months younger. Mild adverse effects like tenderness and swelling were noted but did not significantly impact overall satisfaction. The study concluded that while PRP shows promise in rejuvenating photodamaged skin, further research is necessary to improve preparation methods and optimize results for broader clinical application [156,157,158].

Luiz Charles-de-Sá et al.’s comparative study, in 2020, evaluated the effects of Platelet-Rich Plasma (PRP) and expanded Adipose-Derived Stem Cell (ADSC) therapy on aged human skin. PRP injections induced an inflammatory response that increased collagen fiber deposits and thickened the dermis but did not contribute significantly to tissue regeneration [159,160,161]. In contrast, ADSC therapy led to extracellular matrix (ECM) remodeling, increased production of new elastic fibers, and degradation of elastosis, resulting in significant skin regeneration. The findings suggest that while PRP has limited rejuvenative properties, ADSC therapy shows considerable potential as an effective treatment for skin rejuvenation [162,163].

Randomized trials by Murad Alam et al. highlighted the disparities between the perception of patients and dermatologist assessment. While in 2018 participants perceived areas treated with PRP had significantly improved compared to areas treated with saline, dermatologists saw no apparent differences. These findings are supported by a further experiment, carried out by the same authors, which demonstrated how patient perceptions of PRP’s effects may be stronger than those of objective clinical evaluation. This is a crucial finding for any evaluation of PRP’s effectiveness.

The aim of the clinical study by Paweł Surowiak et al. (2023) 115 was to analyze the effects of PRP on collagen production in human skin, specifically on type I procollagen synthesis. The methodology involved injecting 1 mL of autologous PRP into the skin of each participant’s left forearm, while a placebo (0.9 percent sodium chloride) was injected into the right forearm. The results showed that PRP significantly stimulated the expression of type I procollagen. In contrast, placebo showed minimal collagen production. Interestingly, no correlation was found between patients’ age, sex, or menopausal status and PRP response, although the small sample size may limit these results. No adverse effects were observed.

Finally, in this observational study, Ulusal et al. present the efficacy of Platelet-Rich Plasma treatment combined with hyaluronic acid, demonstrating that Platelet-Rich Plasma treatment significantly improves skin texture and firmness, with quite a high grade of patient satisfaction, especially after three or more treatments. Possible synergistic effects after PRP combination with other regenerative substances should be underscored [121].

In summary, the reviewed articles eloquently point out that PRP is a promising modality for skin rejuvenation since it improves texture, reduces wrinkling, and protects against photoaging.

Anyway, there are some major obstacles that need to be overcome: variability in results, non-uniformity in treatment protocols, and further research that needs to be conducted to define the molecular mechanisms underlying the effects of PRP. This is further put into comparison with other regenerative therapies, such as stem cells and GF; it suggests that PRP, even though it may not be the final answer for skin regeneration, can nevertheless become a useful component in combined treatments. In conclusion, PRP provides some hope in the field of skin rejuvenation but needs to be further researched in order to maximize benefits and improve treatment protocols [164,165].

### 4.2. PRP for Photoaging

Facial rejuvenation is a central theme in esthetic plastic surgery, as is the study of the various mechanisms underlying skin aging. Skin aging is characterized by the degradation of the extracellular matrix (ECM), with loss of elasticity due to the breakdown of fibers such as collagen and elastin and the decrease in fibroblasts. Research of Everts P and Coll. on the treatment of skin aging has explored the use of PRP (Platelet-Rich Plasma), particularly for its GF that support tissue repair. However, the lack of standardized protocols to produce PRP has led to conflicting data [166]. PRP is used in medicine to improve wound healing, with mechanisms parallel to photoaging and skin regeneration; however, the formulation used seems to be decisive on the therapeutic efficacy, as well as its use in combination with other preparations [167,168,169,170,171,172].

Formulations such as PRP buffy coat show superior results thanks to the high concentration of platelets and leukocytes. PRP works via PGF (platelet growth factors) that regulate cell proliferation and ECM remodeling. The use of Pure PRP has shown significant improvements in facial skin after three injections, reducing wrinkles and brown spots, with high patient satisfaction [173]. However, further studies with detailed platelet and white blood cell analyses would be needed to confirm these results [119,173].

A randomized trial by Tsai Y. and Coll. examined the effects of PRP (Platelet-Rich Plasma) and PPP (Platelet-Poor Plasma) on facial rejuvenation, showing significant improvement in periocular wrinkles from 3 to 6 months, with a 20% increase in MFWS (modified Fitzpatrick wrinkle score) in three months [174,175]. PRP did not improve nasolabial folds, while PPP showed a slight, non-significant improvement. PRP improved skin smoothness and texture, while PPP had immediate and long-lasting effects on fine lines, with greater efficacy on dark circles and infraorbital folds [176,177]. PRP-stimulated fibroblasts and modulated inflammation, while PPP acted as a scaffold for the sustained release of GF. Both treatments were safe and well tolerated, with mild and transient adverse events. Limitations included the small sample size and a six-month observation period, indicating the need for further research [109,178].

A study by Ulusal B. has been conducted combining PRP with hyaluronic acid (HA), which showed that PRP can improve skin elasticity by removing photodamaged components and stimulating collagen synthesis. HA, due to its ability to retain water, is used in cosmetic treatments and supports dermal regeneration [179]. Combining PRP and HA, the esthetic results were more satisfactory than the single treatments. Combined treatments reduce pain and improve efficacy due to the viscous environment created by HA [180]. Repeated injections improve facial rejuvenation without surgery [181]. The combination with acupuncture promotes the formation of collagen and elastin, supported by the intake of vitamin C. The injection of PRP and HA after Botox reduces the duration of Botox itself, an effect still to be explained [182]. This confirms the effectiveness of biostimulation with PRP and HA for facial rejuvenation [183,184,185].

The combination of PRP with fractional, ultrapulsed CO_2_ laser treatments seems to reduce laser-related side effects, especially in Asian populations with darker skin, as shown in the study of Hui Q and Coll. PRP can reduce these effects, accelerating healing and improving angiogenesis thanks to its GF, such as VEGF, PDGF, and TGF-b. The synergistic effect between laser and PRP promotes dermal remodeling and epidermal regrowth. Studies have shown that combined treatment is more effective and leads to a faster recovery than laser alone [186,187]. However, high expectations can affect patient satisfaction, especially in more severe cases [188]. PRP is a promising option to improve the efficacy of laser treatment, although further research is needed to optimize clinical protocols [121,189,190].

Although clinical evidence, like in the studies of da Silva L and Coll., seems to show the efficacy of PRP, the lack of standardized protocols for PRP production has led to conflicting data [191,192]. A non-randomized controlled trial compares the use of lyophilized PRP with saline, using objective techniques such as VISIA^®^ imaging to evaluate the effect on photoaging. No significant improvements in collagen levels or skin aging were observed. The results differ from previous studies on fresh PRP, indicating the need for more methodological and well-designed studies [193,194]. The importance of the number of applications and the evaluation period is critical to evaluate the efficacy of lyophilized PRP [195]. Additionally, lyophilization may affect cellular interactions essential for its functionality, which requires further research to fully understand the clinical benefits of lyophilized PRP [196,197,198,199,200,201,202,203].

Our study identified several limitations in using PRP treatments. One key challenge is the variability in PRP preparation, as differences in centrifugation protocols can affect the concentration of platelets and growth factors, leading to inconsistent results. Additionally, many studies had small sample sizes, limiting the generalizability of findings. Patient factors such as age and skin type also influence outcomes but were not always controlled. Although PRP is generally safe, mild side effects like swelling and bruising are common, and rare complications such as infection can occur, especially with improper technique. Furthermore, the lack of standardized protocols for PRP preparation and injection methods makes it difficult to achieve consistent results across studies.

In conclusion, while PRP shows promise, further research is needed to optimize protocols, ensure safety, and address variability in treatment outcomes.

## 5. Conclusions

Autologous platelet concentrates (PCs), such as PRP and PRF, are promising emerging therapies for skin rejuvenation and anti-aging treatments due to their ability to stimulate collagen production, improve skin texture, and promote tissue repair. Among these, PRF stands out for its sustained release of growth factors, potentially offering more stable and lasting results compared to PRP. Additionally, the combination of PRP with complementary treatments, such as hyaluronic acid or laser therapies, enhances its rejuvenation effects. However, significant challenges remain, including variability in outcomes, lack of standardized protocols, and the need for further research to optimize clinical applications. Overall, PCs present significant potential in skin regeneration, but further studies are crucial to refine protocols, establish efficacy, and integrate these therapies into broader regenerative treatment approaches.

## Figures and Tables

**Table 1:** Database search indicators.

Article screening Strategy	Database: Scopus, Web of Science, and PubMed
	Keywords: A “**PRP**”; B “**Facial Aesthetic**”; C “**Photoaging**”; D “**Rejuvenation**”;
	Boolean variable: “AND” and “OR”
	Timespan: 2014–2024
	Language: English

## Data Availability

Data sharing is not applicable to this article as no new data were created or analyzed in this study.

## Abbreviations

ADSC	Adipose-Derived Stem Cell
AGFs	Autologous Growth Factors
APAG	Activated Plasma Albumin Gel
APGF	Autologous Platelet and Growth Factors
BC	Buffy Coat
bFGF	Basic Fibroblast Growth Factor
CGF	Concentrated Growth Factors
ECM	Extracellular Matrix
EGF	Epithelial Growth Factor
GF	Growth Factor
HA	Hyaluronic Acid
I-PRF	Injectable Platelet-Rich Fibrin
LPCGF	Leukocyte and Platelet-Concentrated Growth Factors
MMP	Matrix Metalloproteinases
NaCl	Sodium Chloride
PC	Platelet Concentrates
PDGF	Platelet-derived Growth Factors
PPP	Platelet Poor Plasma
PRF	Platelet-Rich Fibrin
PRGF	Plasma Rich in Growth Factor
PRP	Platelet-Rich Plasma
RBCs	Red Blood Cells
RCF	Relative Centrifugal Force
RP	Red Part
TGF	Transforming Growth Factor
UV	Ultraviolet
VEGF	Vascular Endothelial Growth Factor
WP	White Part
